# On the non‐recursive implementation of multistage sampling without replacement

**DOI:** 10.1016/j.mex.2021.101553

**Published:** 2021-10-20

**Authors:** Philippe Aubry

**Affiliations:** OFB - Office français de la biodiversité - Direction surveillance, évaluation, données - Unité données et appui méthodologique, Saint Benoist, BP 20, F-78612 Le Perray-en-Yvelines, France

**Keywords:** Multistage sampling, Horvitz-Thompson estimator, Expansion estimator, Sen-Yates-Grundy variance, Approximate variance estimator, Self-weighted design, Array data structures, Non-recursive algorithms

## Abstract

Variance estimation in multistage sampling without replacement usually requires considerable computational effort. One option is to implement explicit formulas on a computer, at least for some specific sampling designs. This approach becomes quite cumbersome to handle beyond two stages, both from the formulation and computer implementation points of view. Another option is to provide a general method to compute variance estimates for any number of stages. Such an approach may involve data structures and estimators which are recursively defined.

• The solution we present in this article is intended to be both general and computationally efficient by relying on a full-iterative implementation.
• The definition of the estimators remains implicit as in the recursive approach, but is expressed in terms of recurrence relations translated into iterative algorithms.
• These algorithms rely only on (dense) array data structures. Moreover, most of the necessary computer memory is only used during preliminary steps and is not required when performing the statistical calculations.

## Graphical abstract

**Figure:**
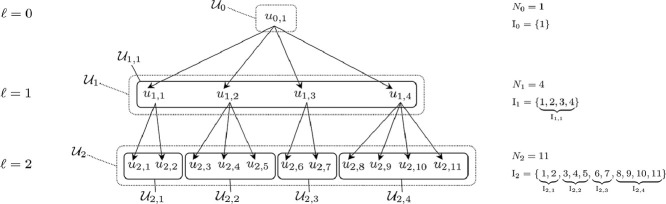

Specifications Table

**Table:** 

Subject Area:	Computational statistics
More specific subject area:	Complex sampling designs; Variance estimation techniques
Method name:	Iterative tree-based computation in multistage sampling without replacement
Name and reference of original method:	D. Bellhouse, Computation of variance-covariance estimates for general multistage sampling designs, in: M. M. Barritt, D. Wishart (Eds.), COMPSTAT 1980: proceedings in computational statistics. 4th symposium held at Edinburgh 1980, Physica-Verlag, Vienna, Austria, 1980, pp. 57–63.
	Bellhouse, Computing methods for variance estimation in complex surveys, J. Off. Stat. (1985) 323–329.
	D. Rylett, D. Bellhouse, TREES: a computer program for complex surveys, in: Proceedings of the survey research methods section, ASA, session XIX, sampling error: methodology, software and applications, American Statistical Association, Alexandria, VA, 1988, pp. 694–697.
Resource availability:	*not applicable*

## Method details

In multistage sampling without replacement, a general method for unbiased variance estimation for the expansion estimator (*i.e.* the extension of the Horvitz-Thompson estimator) suitable for computer implementation was first proposed by Durbin [1], improved by Raj [2] and extended by Rao [3]. For instance, with two-stage sampling, this method consists in estimating the first- and second- stage variance components separately and combining them to form an unbiased estimator of overall sampling variance (*e.g.* [4, sec. 4.3.2] or [5, sec. 7.2.2]). It follows that both the expansion estimator and the sampling variance estimator may be defined recursively which means that the estimator symbol appears on both sides of its definition (*e.g.* [5, p. 158]).

Generally speaking, there is a dichotomy between the recursive definition of a calculation, which is implicit, simple and quick to implement on a computer, and its iterative counterpart, usually more complicated to work out. Owing to the recursive definition of the hierarchical nesting of sampling units and of the sampling variance, it follows that the simplest and most direct way to implement multistage sampling on a computer is to rely on a full recursive implementation, based on a recursively defined data structure and on recursive programming (for recursive programming the reader is referred to Rohl [6] or Rubio-Sánchez [7]).

In imperative programming languages such as FORTRAN, Pascal, C/C++ and so on, the appropriate recursive data structure (a tree) is constructed using dynamically allocated pointers. Recursion is supported by allowing a function (or procedure) to call itself. In this case, recursive calls are automatically managed at the call stack level. For turning a recursive function (or procedure) into an iterative one, one approach is simulating recursion using a manual stack in lieu of relying on the call stack. By using pointers for tree representation and a manual stack for tree traversal we obtain a mixed implementation, that is, recursive for the data structure but iterative for tree traversal (by simulating the recursion). This is precisely what has been proposed by Bellhouse [8,9] and Rylett and Bellhouse [10].

Multistage sampling is also implemented “recursively” in softwares used by some statistical agencies. This is the case for instance of POULPE, a SAS macro program in use at INSEE in France [11], [12]. We guess that the recursion in POULPE is simulated, in a way that has not, however, been detailed in the literature. The main R packages devoted to probability sampling are ‘sampling’ and ‘survey’ [13]. In the ‘sampling’ package [14], to our knowledge variance estimation for multistage sampling is not handled. Conversely, package ‘survey’ [15], [16] estimates the sampling variance by relying on recursive programming (the function ‘multistage’ calls itself). As far as we know this is also the case for the ‘ReGenesees’ package which inherits from ’survey’ regarding multistage sampling [17].

In this technical article we show how multistage sampling can be implemented in a full iterative way, that is without simulating the recursion nor using pointers, by relying on simple (flat) array data structures. Moreover, the solution we propose does not require maintaining in memory all the tree information for performing the statistical computations.

The full-iterative computer implementation detailed here should in particular be useful for handling very large multistage sampling configurations and also for Monte Carlo simulations. It should also be of some pedagogical interest for the in-depth teaching of multistage sampling for a graduate course in computational statistics (even if we have to recognize that this is a somewhat specific technical topic).

Hence, for multistage sampling without replacement, the aim of the present article is to provide (1) a complete recurrent formulation thanks to an appropriate notation and (2) a comprehensive set of algorithms for full-iterative implementation, ensuring efficient statistical computations. To our knowledge, these two technical points have not to date been documented in the statistical computing literature. We focus here on total estimation based on the Horvitz–Thompson estimator, and fixed size designs at all stages. Other estimators can be used without questioning the general organization of the algorithms, and variable size designs can be accommodated in the general case by referring to the Horvitz–Thompson variance instead of the Sen-Yates-Grundy variance (see for instance [18, sec. 7.3] or [4, sec. 4.3.2]).

## Recurrence formulas

In this section, we introduce basic notation conventions that we think appropriate to write recurrence formulas for multistage sampling. Then we express multistage sampling and inclusion probabilities with our notations, and we provide the formulas for estimation: (i) in the general case, that is possibly with unequal inclusion probabilities for all designs, at all stages; (ii) for the particular case of equal probabilities for all designs, at all stages; and finally (iii) for self-weighted sampling.

### Notations

Throughout the formulas section, indices will be noted by a lower case letter, the set of which will be noted by the same letter, but in upper case. For instance, we will have the set of indices $I={\left\{i\right\}}_{i=1}^{N}$ and we will be able to describe the elements indicated by I simply by using set membership, that is i∈I. For the sake of notation simplicity, in what follows we will use i<j∈I in lieu of i<j,i,j∈I.

At each level 0≤ℓ≤L of the tree corresponding to the nested structure of multistage sampling units, the population is denoted $U_{\ell }={\left\{u_{\ell ,i}\right\}}_{i\in I_{\ell }}$ where $I_{\ell }=\left\{1,\ldots ,N_{\ell }\right\}$ is a set of indices (*i.e.* the units labels) as in Daffin et al. [19, p. 512]. The root is at level ℓ=0 in the tree, and will be denoted as the singleton set $U_{0}=\left\{u_{0,1}\right\}$. At levels 0≤ℓ<L, sampling units are populations of units from level ℓ+1. At last level ℓ=L the sampling units are elementary units (that is units which are not further decomposed) which constitute a population of size $N_{L}=N$. Considering clusters of elementary units as ultimate sampling units is merely introducing a level with 100% sampling fraction within the selected clusters. We will denote in a recurrent way:

$$\begin{array}{cccc} \text{for}\;\ell =0 & U_{0}=\left\{u_{0,1}\right\} & N_{0}=1 & I_{0}=\left\{1\right\}\\ \text{for}\;\ell =1,\ldots ,L & U_{\ell }=\underset{i\in I_{\ell -1}}{⋃}U_{\ell ,i} & N_{\ell }=\sum_{i=1}^{N_{\ell -1}}N_{\ell ,i} & I_{\ell }=\left\{1,2,\ldots ,N_{\ell }\right\} \end{array}$$

with $U_{\ell ,i}$ the set of sampling units at level ℓ which constitute the population corresponding to the parent unit $u_{\ell -1,i}$ and $N_{\ell ,i}$ the cardinality of $U_{\ell ,i}$. The index sequence $I_{\ell }$ is partitioned into sub-sequences corresponding to the $N_{\ell -1}$ parent units, that is:

$$I_{\ell }=\left(\underset{I_{\ell ,1}}{\underset{︸}{a_{1},\cdots ,b_{1}}},\underset{I_{\ell ,2}}{\underset{︸}{a_{2},\cdots ,b_{2}}},\cdots ,\underset{I_{\ell ,i}}{\underset{︸}{a_{i},\cdots ,b_{i}}},\cdots ,\underset{I_{\ell ,N_{\ell -1}}}{\underset{︸}{a_{N_{\ell -1}},\cdots ,b_{N_{\ell -1}}}}\right)$$

with $a_{1}=1$, $a_{i+1}=b_{i}+1$ and $b_{i}=\sum_{j=1}^{i}N_{\ell ,j}$ for $1\leq i<N_{\ell -1}$, and finally $b_{N_{\ell -1}}=N_{\ell }$.

**Fig. 1:**
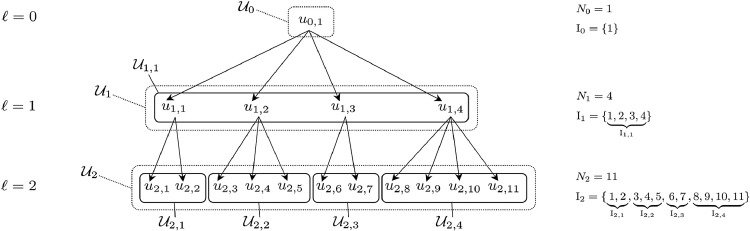
Illustration of our notations for a 2-stage sampling design.

Hence we have a sequence of index sub-sequences $\left(I_{\ell ,1},I_{\ell ,2},\ldots ,I_{\ell ,i},\ldots ,I_{\ell ,N_{\ell -1}}\right)$ which corresponds to the sequence of unit labels for sub-populations $\left(U_{\ell ,1},U_{\ell ,2},\ldots ,U_{\ell ,i},\ldots ,U_{\ell ,N_{\ell -1}}\right)$. For better comprehension, our notations are illustrated in Fig. 1 (for L=2).

### Multistage sampling design

At stage ℓ=0 there is no sampling since by definition the unit $u_{0,1}$ has the probability $\pi_{0,1}=1$ to be included so $s_{0}=\left\{1\right\}$. At stages 1≤ℓ≤L one can apply a sampling design $d_{\ell ,i}$ to each label sub-sequence $I_{\ell ,i}$ associated with the units of the sub-population $U_{\ell ,i}$. This sampling will be denoted by referring to the function $s_{\ell ,i}=d_{\ell ,i}\left(I_{\ell ,i}\right)$ with $s_{\ell ,i}$ a sample of size $n_{\ell ,i}$ ($1<n_{\ell ,i}\leq N_{\ell ,i}$). The multistage sampling design may be written recurrently as:

(1) $$\begin{array}{ccccc} \text{for}\;\ell =0 & & s_{0}=\left\{1\right\} & & \\ \text{for}\;\ell =1,\ldots ,L\;\text{and}\;i\in s_{\ell -1} & & s_{\ell ,i}=d_{\ell ,i}\left(I_{\ell ,i}\right) & & s_{\ell }=\underset{i\in s_{\ell -1}}{⋃}s_{\ell ,i} \end{array}$$

Once the L-stage sampling design is applied, we obtain a sample $s_{L}=s$ including $n_{L}=n$ elementary units for gathering values of the variable of interest y.

The recurrent formulation of multistage sampling allows any design $d_{\ell ,i}$ to be used. Theoretically, the definition of designs at level ℓ>1 may depend on the sample $s_{\ell -1}$. Moreover, samplings at level ℓ>1 among labels $I_{\ell ,i}$ and $I_{\ell ,j}$ for $i\neq j\in s_{\ell -1}$ are not necessarily independent. From this generality it follows that this class of sampling designs may be rather complex. In practice, only a sub-class of multistage sampling designs is used, by imposing both invariance and independence. The invariance property implies that the same design $d_{\ell ,i}$ will be applied to $I_{\ell ,i}$ whatever the sample $s_{\ell -1}$ which includes the parent unit $u_{\ell -1,i}$. The independence property implies that sampling inside each set of labels belonging to the same level ℓ will be applied independently. For more details about invariance and independence, the reader is referred to Särndal et al. [4].

### Inclusion probabilities

With each design $d_{\ell ,i}$ is associated a vector $\pi_{\ell ,i}$ containing $N_{\ell ,i}$ first-order inclusion probabilities :

$$\pi_{\ell ,i}=\left(\pi_{\ell ,a_{i}},\ldots ,\pi_{\ell ,j},\ldots ,\pi_{\ell ,b_{i}}\right)$$

with

$$\sum_{j=a_{i}}^{b_{i}}\pi_{\ell ,j}=n_{\ell ,i}$$

and a matrix $\Pi_{\ell ,i}$ containing $N_{\ell ,i}\times N_{\ell ,i}$ second-order (or joint) inclusion probabilities :

$$\left(\begin{array}{ccccc} \pi_{\ell ,a_{i}a_{i}} & \cdots & \pi_{\ell ,a_{i}j} & \cdots & \pi_{\ell ,a_{i}b_{i}}\\ \vdots & \ddots & \vdots & & \vdots \\ \pi_{\ell ,ja_{i}} & \cdots & \pi_{\ell ,jj} & \cdots & \pi_{\ell ,jb_{i}}\\ \vdots & & \vdots & \ddots & \vdots \\ \pi_{\ell ,b_{i}a_{i}} & \cdots & \pi_{\ell ,b_{i}j} & \cdots & \pi_{\ell ,b_{i}b_{i}} \end{array}\right)$$

with

$$\sum_{j=a_{i}}^{b_{i}}\sum_{k=a_{i}}^{b_{i}}\pi_{\ell ,jk}=n_{\ell ,i}^{2}$$

For $U_{\ell }$ we may denote first-order inclusion probabilities as the sequence of the probability vectors for all sampling designs at level ℓ, and the resulting vector is indexed by indices from $I_{\ell }$, that is :

$$\pi_{\ell }=\left(\pi_{\ell ,1},\pi_{\ell ,2},\ldots ,\pi_{\ell ,i},\ldots ,\pi_{\ell ,N_{\ell -1}}\right)$$

At level ℓ, we denote $\pi_{\ell ,i}^{*}$ the overall (or final) inclusion probability for unit $u_{\ell ,i}$ resulting from the application of sampling designs from level 1 to ℓ. As a result of both invariance and independence for the sampling designs, we get the recurrence :

(2) $$\begin{array}{ccc} \text{for}\;\ell =0\;\text{and}\;j=1 & & \pi_{\ell ,j}^{*}=1\\ \text{for}\;\ell =1,\ldots ,L\text{;}\;i\in I_{\ell -1}\;\text{and}\;j\in I_{\ell ,i} & & \pi_{\ell ,j}^{*}=\pi_{\ell ,j}\pi_{\ell -1,i}^{*} \end{array}$$

In the literature devoted to multistage sampling, this relation is sometimes known as the *selection equation* [20], [21].

### Unequal probability sampling designs at all stages

In this section we consider the recurrence formulation for the general case, allowing unequal inclusions probabilities at all stages, for all sampling designs.

The total may be estimated using an expansion estimator according to the following recurrence:

(3) $$\begin{array}{ccc} \text{for}\;\ell =L\;\text{and}\;i\in s_{\ell } & & {\hat{Y}}_{\ell ,i}=y_{i}\\ \text{for}\;\ell =L,\cdots ,1\;\text{and}\;i\in s_{\ell -1} & & {\hat{Y}}_{\ell -1,i}=\sum_{j\in s_{\ell ,i}}\frac{{\hat{Y}}_{\ell ,j}}{\pi_{\ell ,j}} \end{array}$$

The total estimator is then ${\hat{Y}}_{0,1}$. Its sampling variance may be written using the recurrence:

(4) $$\begin{array}{ccc} \text{for}\;\ell =L\;\text{and}\;i\in I_{\ell } & & V_{\ell ,i}=0\\ \text{for}\;\ell =L,\cdots ,1\;\text{and}\;i\in I_{\ell -1} & & V_{\ell -1,i}=V_{\ell -1,i}^{*}+\sum_{j\in I_{\ell ,i}}\frac{V_{\ell ,j}}{\pi_{\ell ,j}} \end{array}$$

The sampling variance is then $V_{0,1}$. When joint inclusion probabilities can be managed (that is, both calculated and stored), and because we consider only fixed-size designs, the term $V_{\ell -1,i}^{*}$ may be the Sen-Yates-Grundy variance:

(5) $$V_{\ell -1,i}^{*}=\underset{j<k\in I_{\ell ,i}}{\sum \sum }\left(\pi_{\ell ,j}\pi_{\ell ,k}-\pi_{\ell ,jk}\right){\left(\frac{Y_{\ell ,j}}{\pi_{\ell ,j}}-\frac{Y_{\ell ,k}}{\pi_{\ell ,k}}\right)}^{2}$$

The sampling variance estimator may be written using the recurrence:

(6) $$\begin{array}{ccc} \text{for}\;\ell =L\;\text{and}\;i\in s_{\ell } & & {\hat{V}}_{\ell ,i}=0\\ \text{for}\;\ell =L,\ldots ,1\;\text{and}\;i\in s_{\ell -1} & & {\hat{V}}_{\ell -1,i}={\hat{V}}_{\ell -1,i}^{*}+\sum_{j\in s_{\ell ,i}}\frac{{\hat{V}}_{\ell ,j}}{\pi_{\ell ,j}} \end{array}$$

The sampling variance estimator is then ${\hat{V}}_{0,1}$. The term ${\hat{V}}_{\ell -1,i}^{*}$ may be the Sen-Yates-Grundy unbiased variance estimator:

(7) $${\hat{V}}_{\ell -1,i}^{*}=\underset{j<k\in s_{\ell ,i}}{\sum \sum }\left(\frac{\pi_{\ell ,j}\pi_{\ell ,k}-\pi_{\ell ,jk}}{\pi_{\ell ,jk}}\right){\left(\frac{{\hat{Y}}_{\ell ,j}}{\pi_{\ell ,j}}-\frac{{\hat{Y}}_{\ell ,k}}{\pi_{\ell ,k}}\right)}^{2}$$

with $\pi_{\ell ,jk}>0$. At least for high entropy designs, when joint inclusion probabilities cannot be managed, a (biased) variance estimator may be used instead of (7) (see the references cited by Aubry [22]). One possibility is to write [5], [23]:

(8) $${\hat{V}}_{\ell -1,i}^{*}=\sum_{j\in s_{\ell ,i}}\frac{c_{\ell ,j}}{\pi_{\ell ,j}^{2}}{\left({\hat{Y}}_{\ell ,j}-{\hat{Y}}_{\ell ,j}^{*}\right)}^{2}$$

with

(9) $${\hat{Y}}_{\ell ,j}^{*}=\pi_{\ell ,j}\;\frac{\sum_{m\in s_{\ell ,i}}c_{\ell ,m}\frac{{\hat{Y}}_{\ell ,m}}{\pi_{\ell ,m}}}{\sum_{m\in s_{\ell ,i}}c_{\ell ,m}}$$

and $c_{\ell ,j}=\left(1-\pi_{\ell ,j}\right)n_{\ell ,i}/\left(n_{\ell ,i}-1\right)$ for $j\in s_{\ell ,i}$.

When all sampling designs are PPSWOR (*Probability Proportional to Size WithOut Replacement*), then the size is computed at all levels 1≤ℓ<L from the values defined for the population of elementary units. For $i\in I_{\ell }$ and ℓ=L, we denote $x_{i}$ the value for the size-variable, associated with the elementary unit $u_{\ell ,i}$. The total $\sum x_{i}$, can be decomposed by the following recurrence:

(10) $$\begin{array}{ccc} \text{for}\;\ell =L\;\text{and}\;i\in I_{\ell } & & X_{\ell ,i}=x_{i}\\ \text{for}\;\ell =L,\ldots ,1\;\text{and}\;i\in I_{\ell -1} & & X_{\ell -1,i}=\sum_{j\in I_{\ell ,i}}X_{\ell ,j} \end{array}$$

The total is then $X_{0,1}$.

### Equal probability sampling designs at all stages

When every population $U_{\ell ,i}$ is sampled using SRSWOR (*Simple Random Sampling WithOut Replacement*) of size $n_{\ell ,i}$ out of $N_{\ell ,i}$ ($1<n_{\ell ,i}\leq N_{\ell ,i}$), then formulas become considerably simpler. Indeed, up to second order, inclusion probabilities are simply (for $j\neq k\in I_{\ell ,i}$):

(11) $$\begin{array}{c} \pi_{\ell ,j}=\frac{n_{\ell ,i}}{N_{\ell ,i}}\\ \pi_{\ell ,jk}=\frac{n_{\ell ,i}\left(n_{\ell ,i}-1\right)}{N_{\ell ,i}\left(N_{\ell ,i}-1\right)} \end{array}$$

It follows that the recurrence for the total estimator is now written:

(12) $$\begin{array}{ccc} \text{for}\;\ell =L\;\text{and}\;i\in s_{\ell } & & {\hat{Y}}_{\ell ,i}=y_{i}\\ \text{for}\;\ell =L,\ldots ,1\;\text{and}\;i\in s_{\ell -1} & & {\hat{Y}}_{\ell -1,i}=\frac{N_{\ell ,i}}{n_{\ell ,i}}\sum_{j\in s_{\ell ,i}}{\hat{Y}}_{\ell ,j} \end{array}$$

The recurrence for sampling variance is now:

(13) $$\begin{array}{ccc} \text{for}\;\ell =L\;\text{and}\;i\in I_{\ell } & & V_{\ell ,i}=0\\ \text{for}\;\ell =L,\ldots ,1\;\text{and}\;i\in I_{\ell -1} & & V_{\ell -1,i}=N_{\ell ,i}^{2}\left(1-\frac{n_{\ell ,i}}{N_{\ell ,i}}\right)\frac{S_{\ell ,i}^{2}}{n_{\ell ,i}}+\frac{N_{\ell ,i}}{n_{\ell ,i}}\sum_{j\in I_{\ell ,i}}V_{\ell ,j} \end{array}$$

with

$$S_{\ell ,i}^{2}=\frac{1}{N_{\ell ,i}-1}\sum_{j\in I_{\ell ,i}}{\left(Y_{\ell ,j}-{\bar{Y}}_{\ell ,i}\right)}^{2}\;\text{and}\;\;{\bar{Y}}_{\ell ,i}=\frac{1}{N_{\ell ,i}}\sum_{j\in I_{\ell ,i}}Y_{\ell ,j}$$

and accordingly, the recurrence for the unbiased sampling variance estimator is:

(14) $$\begin{array}{ccc} \text{for}\;\ell =L\;\text{and}\;i\in s_{\ell } & & {\hat{V}}_{\ell ,i}=0\\ \text{for}\;\ell =L,\ldots ,1\;\text{and}\;i\in s_{\ell -1} & & {\hat{V}}_{\ell -1,i}=N_{\ell ,i}^{2}\left(1-\frac{n_{\ell ,i}}{N_{\ell ,i}}\right)\frac{{\hat{S}}_{\ell ,i}^{2}}{n_{\ell ,i}}+\frac{N_{\ell ,i}}{n_{\ell ,i}}\sum_{j\in s_{\ell ,i}}{\hat{V}}_{\ell ,j} \end{array}$$

with

$${\hat{S}}_{\ell ,i}^{2}=\frac{1}{n_{\ell ,i}-1}\sum_{j\in s_{\ell ,i}}{\left(Y_{\ell ,j}-{\hat{\bar{Y}}}_{\ell ,i}\right)}^{2}\;\text{and}\;\;{\hat{\bar{Y}}}_{\ell ,i}=\frac{1}{n_{\ell ,i}}\sum_{j\in s_{\ell ,i}}Y_{\ell ,j}$$

### Self‐weighted multistage sampling

With our notations, the weight of an elementary unit $u_{\ell ,i}$ is $w_{\ell ,i}=1/\pi_{\ell ,i}^{*}$ for $i\in I_{\ell }$ and ℓ=L. A design is self-weighted when all elementary units have the same weight that is, for ℓ=L, when $\pi_{\ell ,i}^{*}=\pi_{\ell ,j}^{*}$ ($i\neq j\in I_{\ell })$. For the sake of simplicity, this common inclusion probability will be denoted $\pi^{*}$.

Let $X_{\ell ,i}$ be the size of the sampling unit $u_{\ell ,i}$ in number of underlying elementary units. We have the following definition by recurrence:

(15) $$\begin{array}{ccccc} \text{for}\;\ell =L\;\text{and}\;i\in I_{\ell } & & X_{\ell ,i}=1 & & \\ \text{for}\;\ell =L,\ldots ,1\;\text{and}\;i\in I_{\ell -1} & & X_{\ell -1,i}=\sum_{j\in I_{\ell ,i}}X_{\ell ,j} & \end{array}$$

Obtaining a self-weighted L-stage sampling design requires the selection of samples of constant size $m_{\ell }$ among units $U_{\ell ,i}$ ($i\in I_{\ell -1}$) for ℓ=1,…,L with a probability proportional to their size, that is:

(16) $$\text{for}\;\ell =1,\cdots ,L\text{;}\;i\in I_{\ell -1}\;\text{and}\;j\in I_{\ell ,i}\pi_{\ell ,j}=m_{\ell }\frac{X_{\ell ,j}}{\sum_{k\in I_{\ell ,i}}X_{\ell ,k}}$$

Applying recurrence (2), it follows that the constant overall inclusion probability for elementary units is equal to $\pi^{*}=n/N$ with:

(17) $$n=\prod_{\ell =1}^{L}m_{\ell }$$

At last stage L, expression (16) shows that it is necessary to include elementary units with the following probability:

(18) $$\text{for}\;\ell =L\text{;}\;i\in I_{\ell -1}\;\text{and}\;j\in I_{\ell ,i}\pi_{\ell ,j}=\frac{m_{\ell }}{N_{\ell ,i}}$$

which does not depend on j. In the most general case, for ℓ=1,…,L, cardinalities $N_{\ell ,i}$ ($i\in I_{\ell -1}$) differ, and therefore sizes $X_{\ell ,j}$ ($j\in I_{\ell ,i}$) also differ. In this case, units at level 1≤ℓ<L need to be selected using PPSWOR with inclusion probabilities (16) whilst elementary units have to be selected using SRSWOR with probabilities (18). Although the last stage is SRSWOR we rely here on the general formulation.

In the simplest case, for ℓ=1,…,L cardinalities are equal, that is, $M_{\ell }=N_{\ell ,i}$ ($i\in I_{\ell -1}$), and therefore sizes $X_{\ell ,j}=N/N_{\ell }$ ($j\in I_{\ell ,i}$) are also equal and the same arises for first-order inclusion probabilities $\pi_{\ell ,j}=m_{\ell }/M_{\ell }$ ($j\in I_{\ell ,i}$) with $1<m_{\ell }\leq M_{\ell }$. Applying recurrence (2) it follows, as expected:

(19) $$\pi^{*}=\frac{\prod_{\ell =1}^{L}m_{\ell }}{\prod_{\ell =1}^{L}M_{\ell }}=\frac{n}{N}$$

In this limiting case, sampling units are selected by SRSWOR at each stage with probabilities $\pi_{\ell ,j}=m_{\ell }/M_{\ell }$.

## Algorithms

In this section we provide algorithms for full-iterative computer implementation of multistage sampling. The principle that guided algorithm elaboration is that the tree corresponding to the hierarchical nesting of sampling units is represented by arrays of which only a part remains useful for statistical computations. Hence, the major part of the required computer memory is used only temporarily and can be released as soon as the preliminary steps are achieved. Moreover, no sparse arrays are used so that no memory places are wasted.

Although we do not provide a theoretical analysis of time and space complexity of the proposed algorithms — a topic far beyond the scope of this paper — we are confident in their efficiency, especially when implemented in a compiled programming language usually used for statistical computing, such as C/C++ or FORTRAN (possibly for interoperating with the R programming environment).

### Notations

Algorithms (procedures or functions) are given as pseudocode, with classical control structures. There are three possible passing modes for a procedure parameter depending on whether it is only on input (“in” mode; its value remains unchanged), only on output (“out” mode; its value is defined within the procedure) or both on input/output (“in-out” mode; its value is modified within the procedure). We put a down-arrow, an up-arrow or a down-up-arrow above a parameter’s symbol for specifying in, out, and in-out passing modes, respectively. By definition, all parameters of a function are in input-only passing mode and therefore down-arrows will be omitted.

### Hierarchical nesting of units

**Fig. 2:**
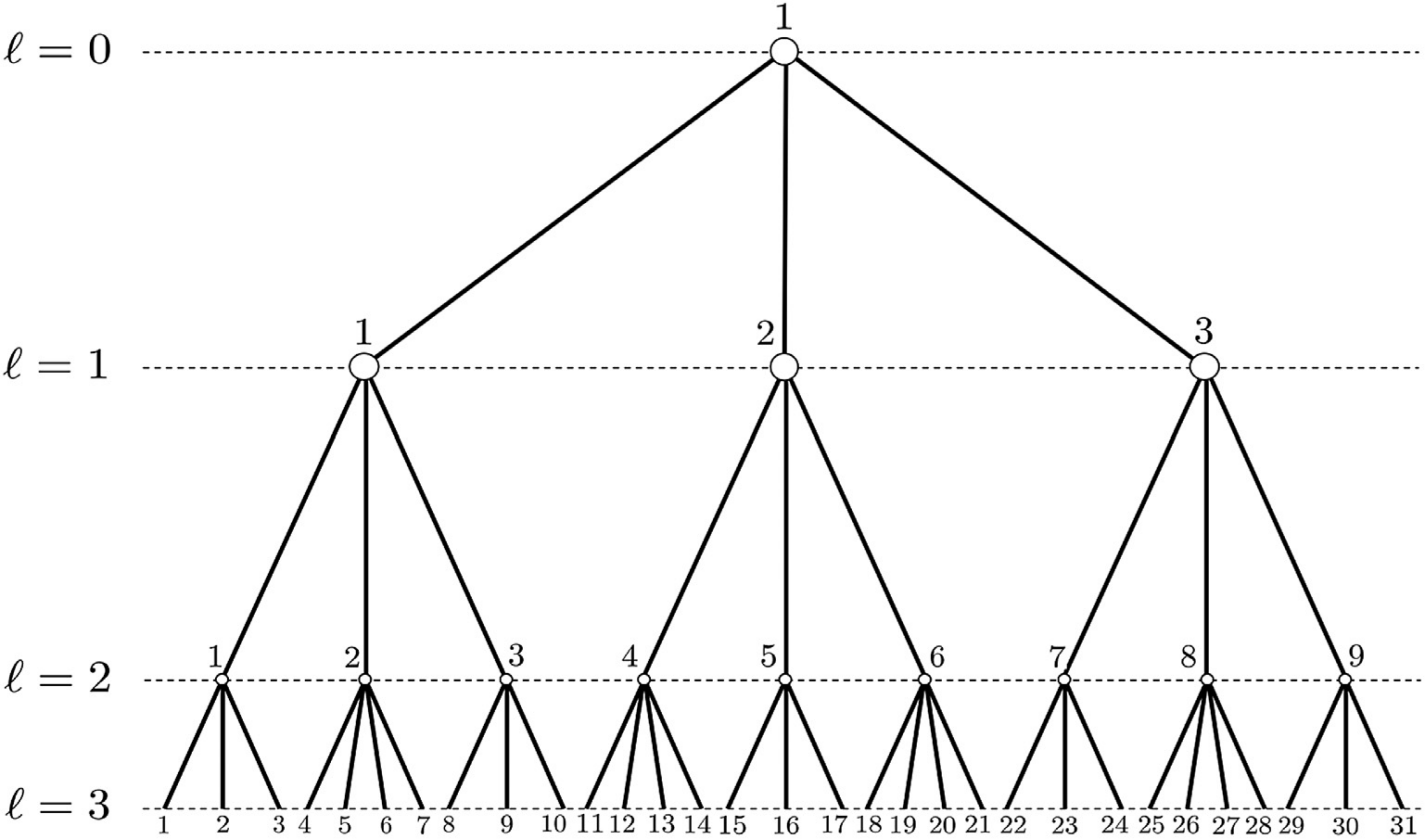
Example of tree for a 3-stage sampling design.

The hierarchical nesting of units leads to a tree whose nodes correspond to sampling units (primary sampling units, secondary sampling units and so forth until elementary sampling units). Without loss of generality, we illustrate the tree structure involved by multistage sampling with a small example for L=3 (Fig. 2).

**Fig. 3:**
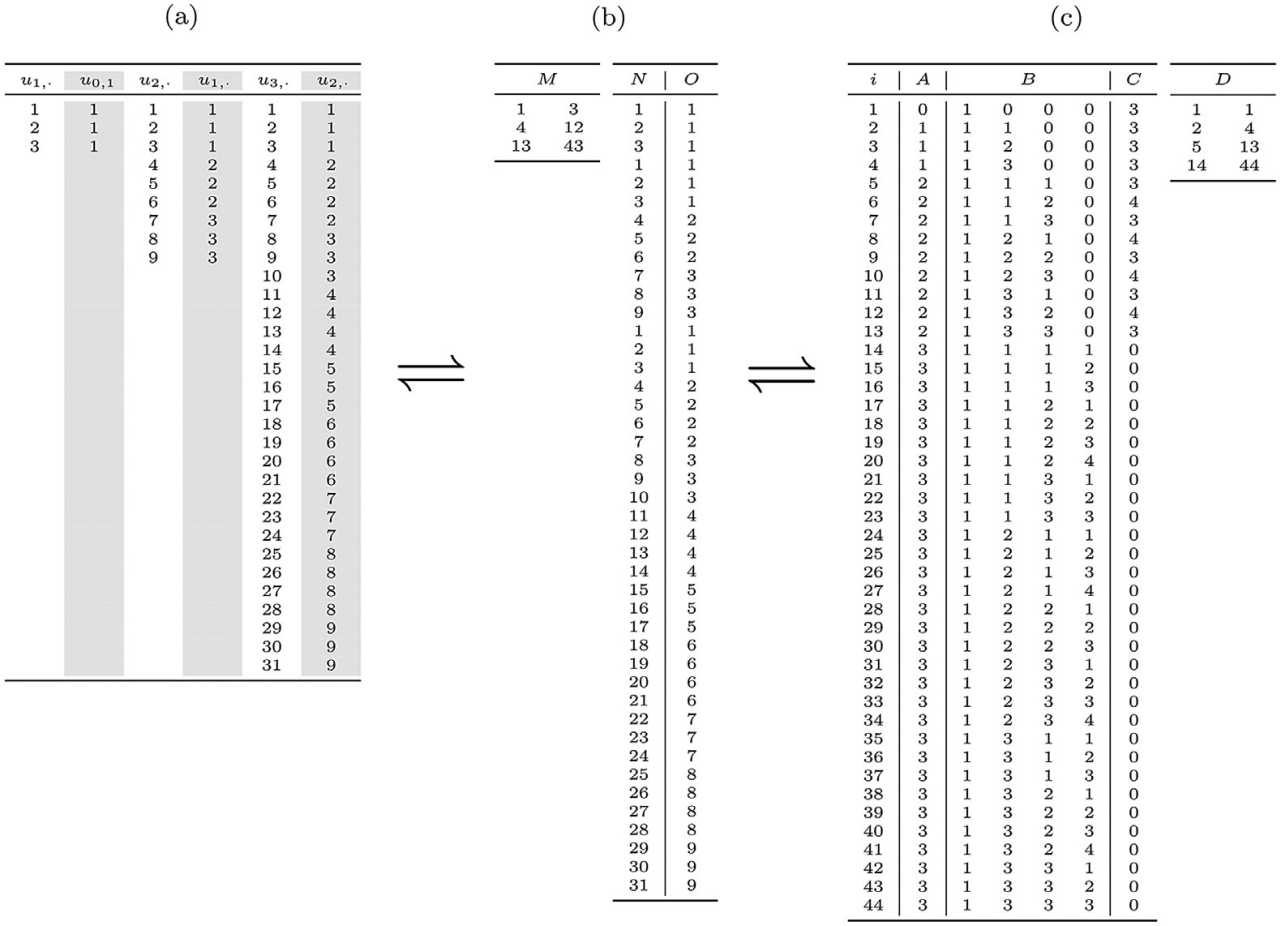
Three equivalent array-based representations for the tree used as an example. (a) Dataset as a sparse array. (b) dataset as dense arrays. (c) Array representation of the tree.

In practice the tree information is given by the data. All the needed information is a label for each unit for levels ℓ=1,⋯,L, as well as the membership of each unit of level ℓ within a unit of level ℓ−1. The data corresponding to our example are illustrated in Fig. 3(a).

The same data can be stored as shown in Fig. 3(b), with an access array M containing indices of arrays N and O for delimiting the levels, array N containing the unit labels, and array O containing the membership information, that is, the label of the parent unit.

For performing statistical computations we deal with in this article, at first glance it may seem necessary to access in memory the tree path for all nodes. This is for instance the case for a recursive implementation such as that of Bellhouse [8,9] and Rylett and Bellhouse [10]. In this paper, not only do we not convert the data into a recursive data structure for representing the corresponding tree (example in Fig. 2), but moreover we propose a solution where paths are used only at a preliminary step.

We manipulate a set of four arrays: (i) A containing the level of each node (between 0 for the root and L for the leafs); (ii) B containing the paths for each node; (iii) C containing the number of nodes’ children; (iv) D for efficient node browsing at a given level. Sorting the arrays A, B and C is a prerequisite for subsequent treatments.

The conversion from arrays M and O describing the dataset is ensured by Algorithm 1 which returns (unsorted) arrays A and B. Note that the 0-filling step at the end of the algorithm is irrelevant when computer implementation is done in a programming language where arrays are automatically initialized with 0 or when a specific syntax exists for doing such initialization in fewer instructions. Of course it is possible to convert (sorted) arrays A, B describing the tree into a dataset using the reverse conversion Algorithm 2 which returns arrays M, N and O.

**Algorithm 1:**
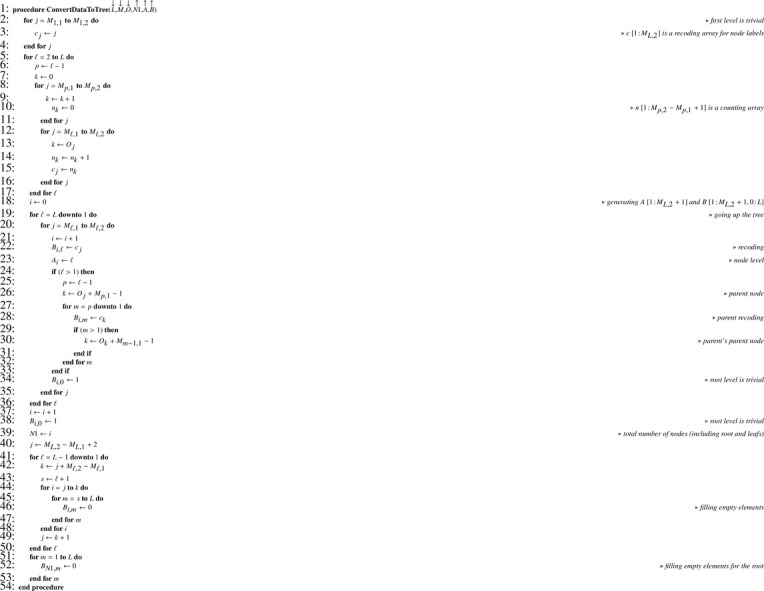
Converting a dataset to the corresponding tree.

**Algorithm 2:**
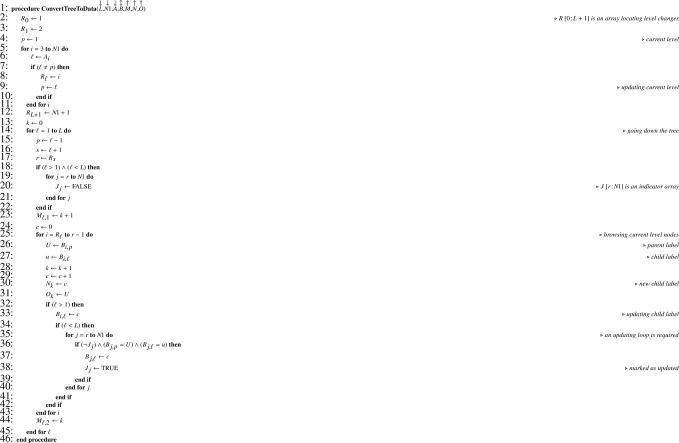
Converting a tree to the corresponding dataset.

Information contained in arrays A and B allows generating a reordering index T according to Algorithm 3. In the latter, note that the choice of the sorting algorithm is left to the discretion of the reader. Thanks to T, arrays A and B can be reordered in place using Algorithm 4. For this procedure, an upper bound (UB) lower than the lower bound (LB) for the number of columns indicates that the array to be reordered (G) has one dimension only. Once the reordering of A and B is achieved, access array D is built using Algorithm 5. Note that as soon as D is built, regarding the total number of nodes N1 we have of course $N1=D_{L,2}$. Hence, when D is a parameter, it is not necessary to also pass N1. Finally, the number of children for each node can be computed according to Algorithm 6. For our example, the set of arrays A, B, C and D is illustrated in Fig. 3(c). Note that arrays A and B are necessary for obtaining C in proper order and for building D according to the simple Algorithm 5, but later on they are no longer useful for statistical computations and corresponding memory may be released.

**Algorithm 3:**
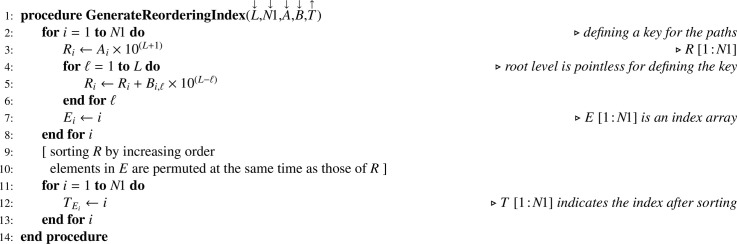
Generating a reordering index from tables of levels and paths.

**Algorithm 4:**
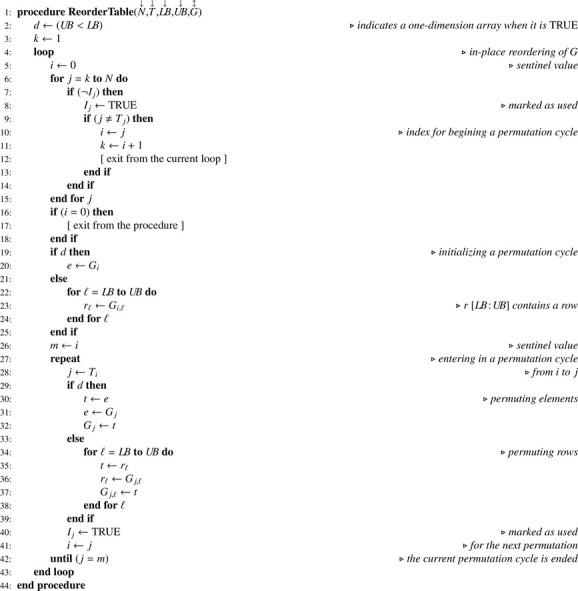
In-place reordering of array G.

**Algorithm 5:**
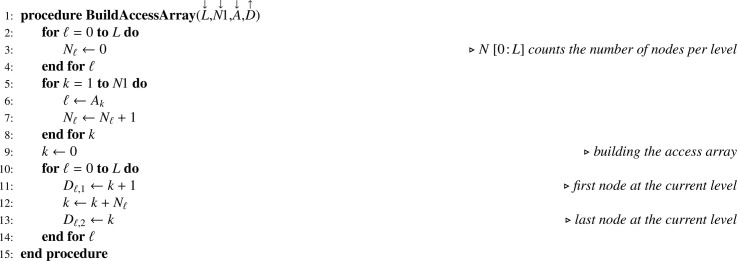
Building an array for browsing nodes at a given level.

**Algorithm 6:**
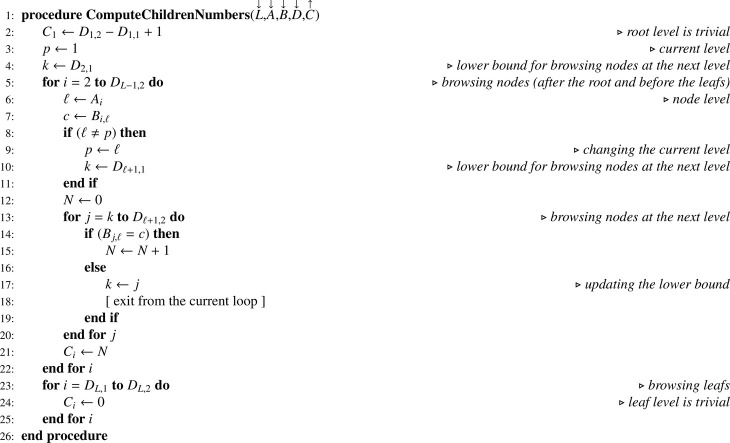
Computing the number of children for all nodes.

For testing or Monte Carlo simulation purpose it is convenient to be able to automatically generate the hierarchical nesting of units for any number of stages. This is what Algorithm 7 does. In this paper, this algorithm is the only one which simulates the recursion associated with a tree. According to the number of stages L and an interval for random population sizes (*MIN, MAX*), it returns the total number of nodes in the tree (N1), and arrays A, B and C. Next, Algorithms 3 and 4 have to be used before executing Algorithm 5. After that, as previously, memory for arrays A and B may be released.

**Algorithm 7:**
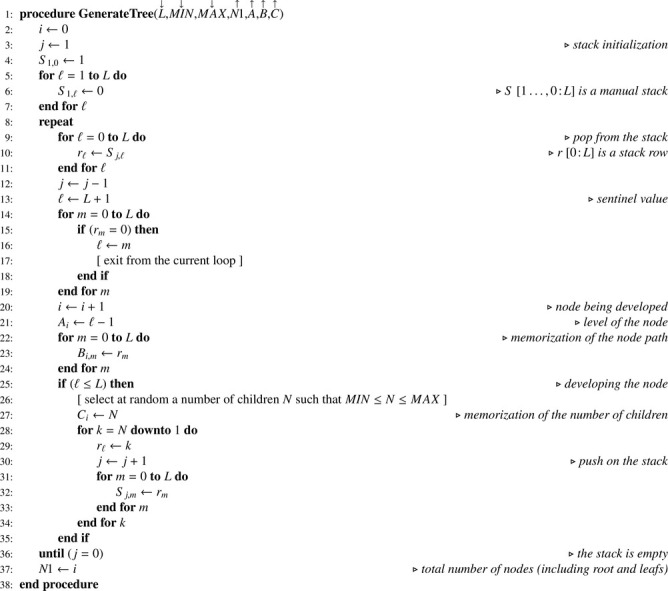
Generating hierarchical nesting of units on L levels.

### Multistage sampling without replacement

Let S be a one-dimension array containing samples sizes associated with nodes at levels 1≤ℓ≤L. Multistage sampling without replacement as formalized by recurrence formula (1) may be implemented using Algorithm 8 which generates an indicator array I. As a great diversity of sampling designs may be used, details are left to the discretion of the reader. When PPSWOR is used, array TX must be computed beforehand using Algorithm 9 — which is a mere translation of recurrence formula (10) — where X is a one-dimension array corresponding to a size variable x.

**Algorithm 8:**
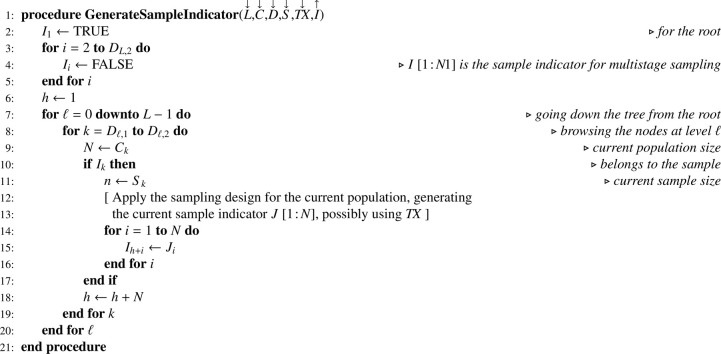
Selecting a sample using multistage sampling without replacement.

**Algorithm 9:**
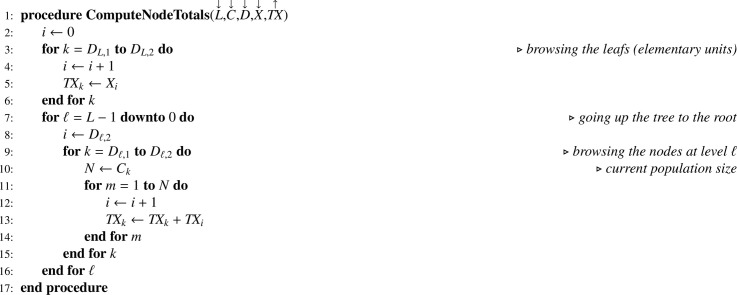
Computing the total for all nodes in the tree.

### Statistical computations for SRSWOR at all stages

Let Y be the array corresponding to the variable of interest y. In a Monte Carlo simulation, as y is known for the totality of the elementary units, Algorithm 9 applied to Y returns TY. Then it is possible to apply recurrence formula (13), translated into Algorithm 10.

**Algorithm 10:**
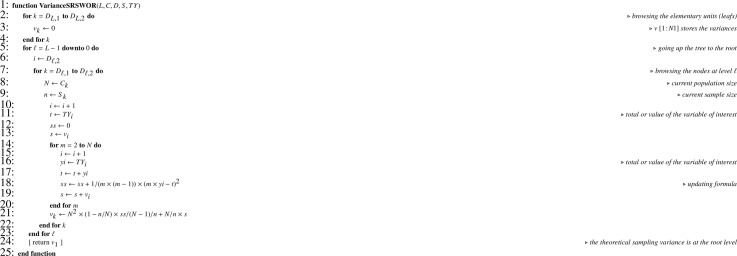
Theoretical sampling variance when all sampling designs are SRSWOR.

In practice Y contains missing values for the elementary units not in the final sample. Recurrence formulas (12) and (14) translate into Algorithms 11 and 12, respectively, where the array I corresponds to the sample indicator.

**Algorithm 11:**
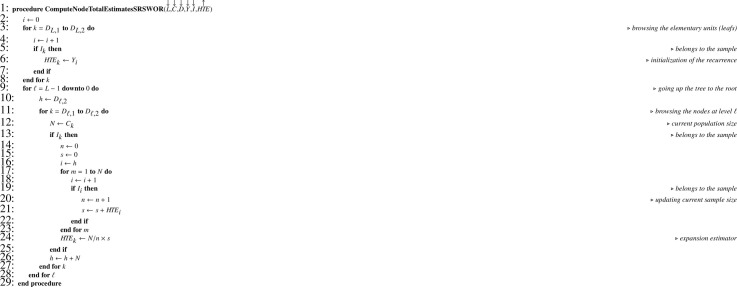
Computing total estimates when all sampling designs are SRSWOR.

**Algorithm 12:**
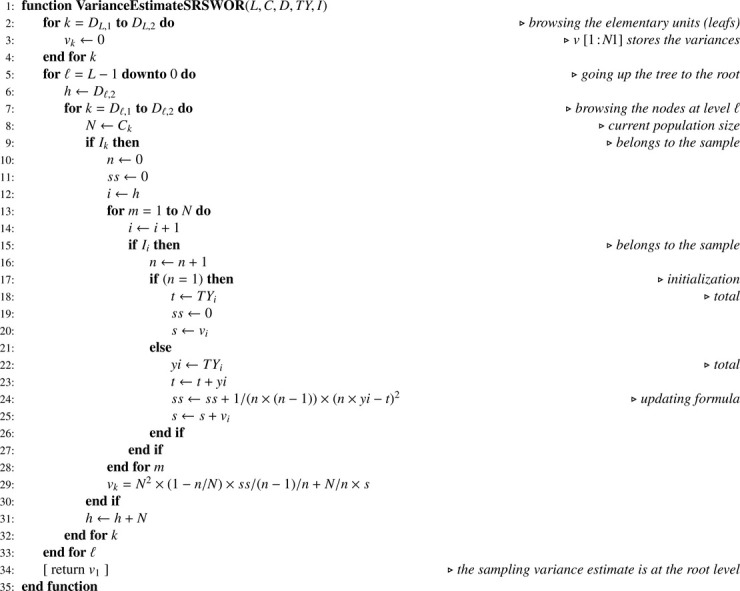
Sampling variance estimate when all sampling designs are SRSWOR.

Note that, in Algorithms 10 and 12, one-pass computation of the sum of squared deviations (ss) is performed using the numerically stable updating formula proposed by Youngs and Cramer [24] (see also [25, sec. 2.4.4]).

### Statistical computations for PPSWOR at all stages

Let X be the one-dimension array corresponding to size variable x, positively correlated with the variable of interest y. First, Algorithm 9 is applied to X for returning TX. Next, unequal inclusion probabilities have to be stored. First-order inclusion probabilities are obviously stored in a one-dimension array (P). For computing overall first-order inclusion probabilities ($P^{*}$), recurrence formula (2) translates into Algorithm 13.

**Algorithm 13:**
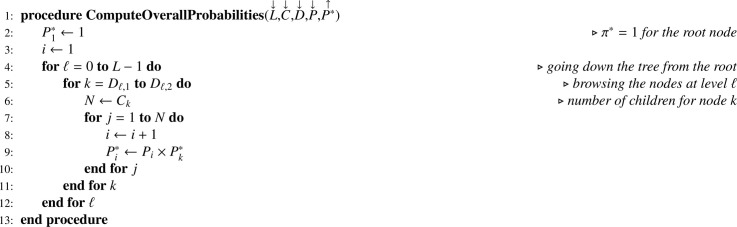
Computing overall first-order inclusion probabilities.

When joint inclusion probabilities can be managed, they have to be stored in an efficient way, that is in a one-dimension array. For the complete set of units involved in a multistage sampling situation, the matrix of joint inclusion probabilities is block diagonal. Owing to the symmetry of joint inclusion probabilities and to the block diagonal structure, it turns out that a very efficient storage requires only two one-dimension arrays: (i) a small one (F) for indicating submatrix changes; (ii) a larger one (PP) to store all joint inclusion probabilities (without duplication). These arrays are generated using Algorithm 14. When joint inclusion probabilities cannot be managed, F and PP arrays are irrelevant, and Algorithm 14 has to be simplified accordingly (this straightforward modification if left to the reader). Since a great diversity of sampling designs may be used, parameters required for computing Π matrices are omitted and details are left to the discretion of the reader. Finally, retrieving joint inclusion probabilities needs Algorithm 15 which, in turn, relies on Algorithm 16.

**Algorithm 14:**
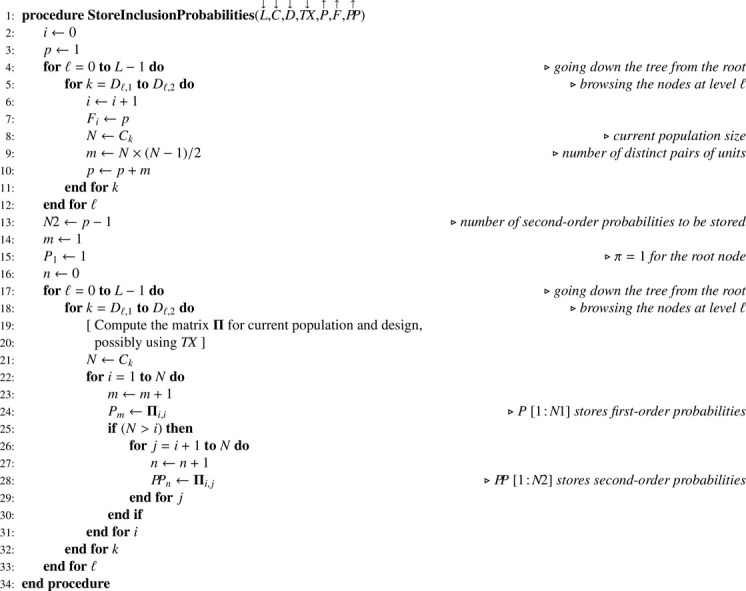
Storage of first- and second-order inclusion probabilities.

**Algorithm 15:**
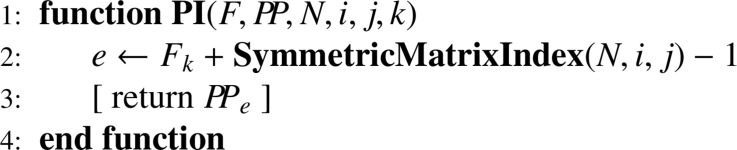
Returning the joint inclusion probability for i≠j in the subpopulation of size N corresponding to the node k.

**Algorithm 16:**
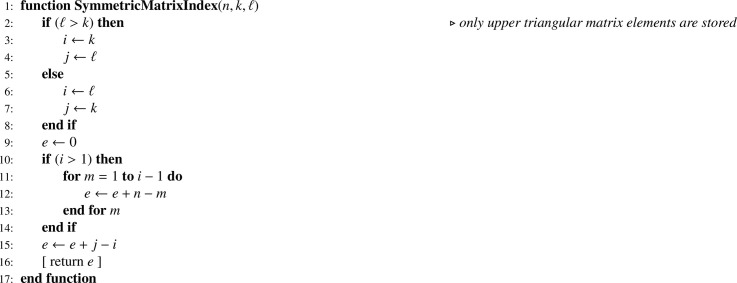
Returning the index for an element in a symmetric matrix which upper triangular part is stored in an one-dimension array.

When Monte Carlo simulations are performed, computing the theoretical sampling variance in the general case (that is, with unequal inclusion probabilities) requires recurrence formula (4) translated into Algorithm 17. Exact computation needs formula (5) (Sen-Yates-Grundy variance) which translates into Algorithm 18 and returns array V required by Algorithm 17.

Unbiased estimation of the population total is achieved using recurrence formula (3), translated into Algorithm 19. Estimating the sampling variance requires recurrence formula (6) translated into Algorithm 20. When joint inclusion probabilities can be managed, node variance estimates can be computed using formula (7), translated into Algorithm 21 returning V which is a parameter of Algorithm 20. Otherwise, one possibility is to use approximation (8) translated into Algorithm 22, which only requires first-order inclusion probabilities.

## Method validation

This section is aimed at providing both numerical illustration and control for the validity of statistical computation algorithms proposed in this article.

Generating artificial multistage sampling configurations for any number of stages (Algorithm 7) is a very convenient way for applying and validating all algorithms proposed in this article on very large examples. Nevertheless, there is no generality loss by referring to the small example used throughout this paper, with L=3 stages and MIN=3 or MAX=4 children for each node at levels 1≤ℓ<L.

Self-weighted multistage sampling design is a good candidate for testing purpose since it involves unequal probability sampling. Besides, the self-weighting property is very easy to check thanks to selection equation (2); an error in this regard would immediately indicate an implementation error. As PPSWOR design we refer here to the Hanurav-Vijayan procedure which is appropriate, provided it is correctly implemented [22]. For the sake of completeness, we also provide the explicit expression of the theoretical sampling variance (20), which translates into Algorithm 23. This example shows how cumbersome the expression of the variance becomes when the number of stages increases. Terms $s_{1,1}$, $s_{1,2}$, $s_{2,1}$ and $s_{2,2}$ are indicated to easily identify their counterparts in Algorithm 23.

(20) $$\begin{array}{cc} V(\hat{Y})\;\;\;\;\;\;\; & =\underset{s_{1,1}}{\underset{︸}{{\sum \sum }_{i<j\in I_{1}}\left(\pi_{1,i}\pi_{1,j}-\pi_{1,ij}\right){\left(\frac{Y_{1,i}}{\pi_{1,i}}-\frac{Y_{1,j}}{\pi_{1,j}}\right)}^{2}}}\\ \;\;\;\;\;\;\; & +\underset{s_{1,2}}{\underset{︸}{\sum_{i\in I_{1}}\frac{1}{\pi_{1,i}}\left[{\underset{︸}{{\sum \sum }_{k<\ell \in I_{2,i}}\left(\pi_{2,k}\pi_{2,\ell }-\pi_{2,k\ell }\right){\left(\frac{Y_{2,k}}{\pi_{2,k}}-\frac{Y_{2,\ell }}{\pi_{2,\ell }}\right)}^{2}}}_{s_{2,1}}+{\underset{︸}{\sum_{k\in I_{2,i}}\frac{1}{\pi_{2,k}}\left[N_{3,k}^{2}\left(1-\frac{n_{3,k}}{N_{3,k}}\right)\frac{S_{3,k}^{2}}{n_{3,k}}\right]}}_{s_{2,2}}\right]}} \end{array}$$

**Algorithm 17:**
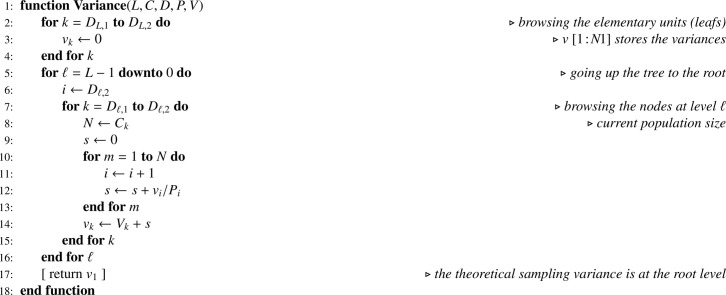
Theoretical sampling variance in the general case.

**Algorithm 18:**
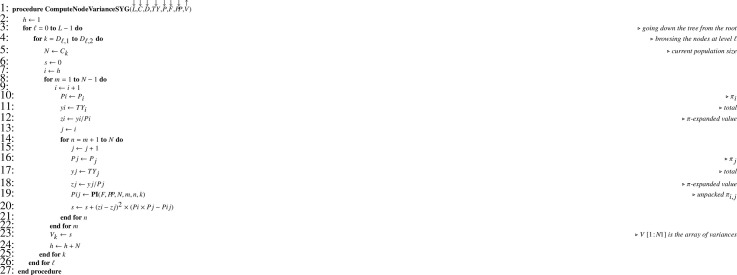
Computing node variances (Sen-Yates-Grundy).

**Algorithm 19:**
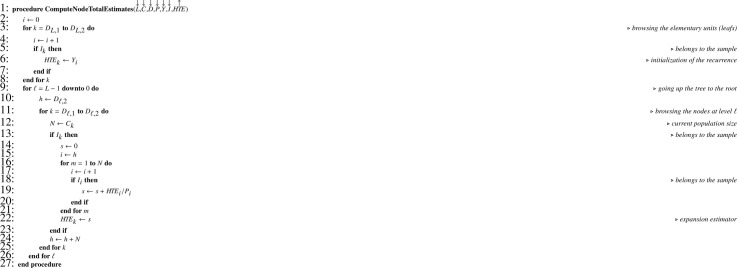
Computing total estimates in the general case.

**Algorithm 20:**
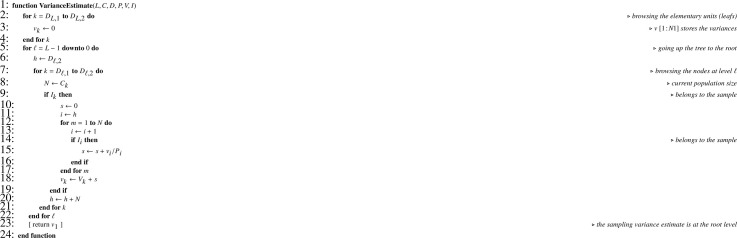
Sampling variance estimate in the general case.

**Algorithm 21:**
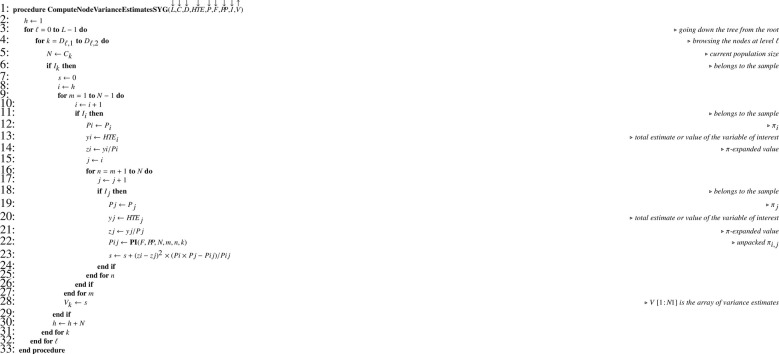
Computing Sen-Yates-Grundy variance estimates.

**Algorithm 22:**
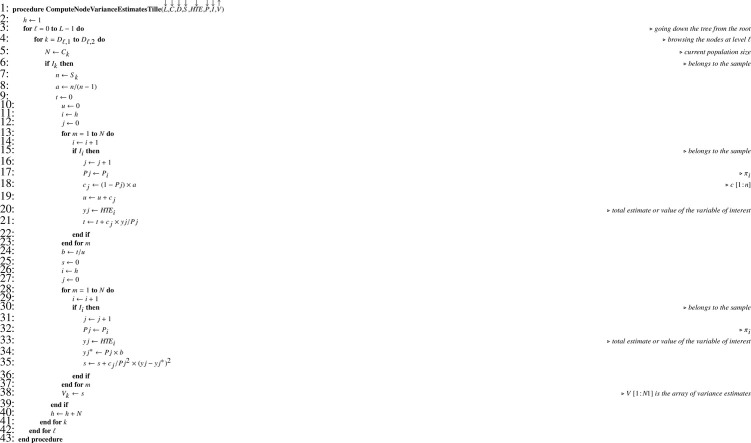
Computing approximate variance estimates.

**Fig. 4:**
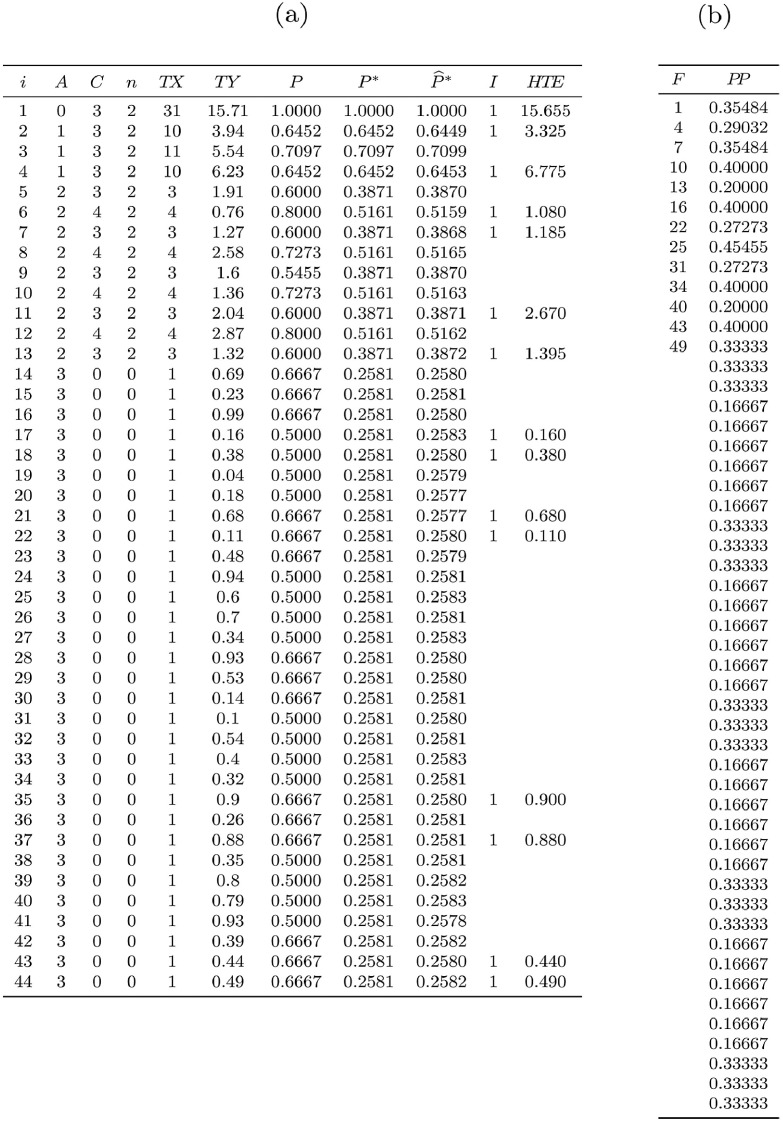
Numerical illustration for the 3-stage self-weighted sampling design used as an example. (a) Summary table with some data and results: i - node index; A - node level; C - children number; n - size sample; TX - node size; TY - node total; P - inclusion probability, $P^{*}$ - overall inclusion probability; ${\hat{P}}^{*}$ - Monte Carlo estimate of $P^{*}$ ($10^{7}$ replications); HTE - expansion estimator. (b) Array representation of second-order inclusion probabilities: F - index indicating submatrix changes; PP - joint inclusion probabilities.

Theoretical first-order (P) and overall ($P^{*}$) inclusion probabilities as well as estimates of the latter (${\hat{P}}^{*}$) obtained after $10^{7}$ replications of sampling [22, sec. 4.1] are given in Fig. 4(a). An example of sample is given in column I (sample indicator) and the result of procedure 19 appears in column HTE (the total estimate is $\hat{Y}=15.655$). The sampling variance computed by function 17 is $V(\hat{Y})=7.875$. As expected this is also the value obtained using Algorithm 23. We can estimate the sampling variance using either Sen-Yates-Grundy variance estimator (7) (case 1) or approximation (8) (case 2). In the former case, computations require second-order inclusion probabilities given in Fig. 4(b). Sampling variance estimates for the sample given in 4(a) are $\hat{V}\left(\hat{Y}\right)=18.945$ for case 1 and $\hat{V}\left(\hat{Y}\right)=13.931$ for case 2. As expected, the Monte Carlo estimate for the sampling variance is close to its theoretical value since we obtain 7.870. Finally, the Monte Carlo estimate for the expectation of $\hat{V}\left(\hat{Y}\right)$ is 7.873 for case 1 — again very close to the theoretical value — and 7.652 for case 2, which indicates a slight underestimation due to the approximation, and illustrates that estimator (8) tends to be down-biased.

**Algorithm 23:**
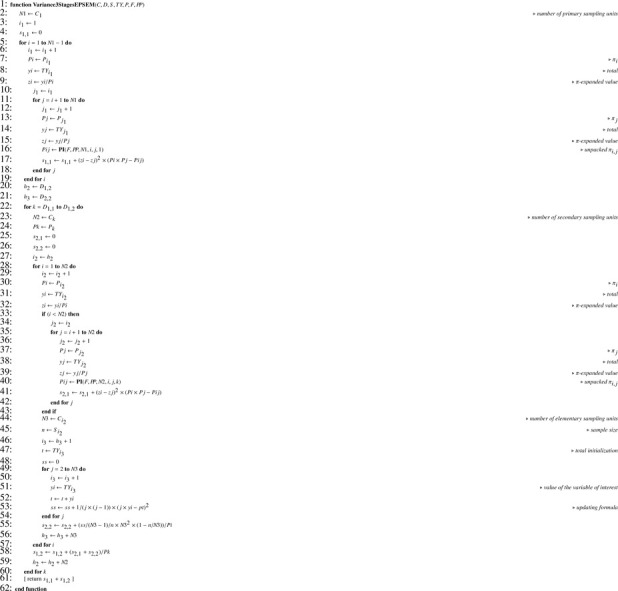
Explicit variance computation for self-weighted three-stage sampling.

## Additional information

In practice, two-stage and three-stage sampling designs are rather common, and four-stage sampling is not exceptional. Conversely, multistage sampling beyond five stages seems not to be used.

When sampling geographical space, multistage sampling is very useful from a logistical point of view for reducing the cost of travelling between the elementary spatial sampling units. It follows that, in environmental sciences for instance, multistage sampling is part of the toolbox for designing large-scale natural resource surveys, often in combination with stratification. The multistage sampling of nested spatial sampling units also meets the concern of being able to study a phenomenon at several spatial scales.

The data collected on the elementary units are not always devoted only to estimating finite population parameters, and may rather be seen as all-purpose data. As such, their analysis and modeling should be easy, which implies they all have the same weight, that is, the same overall inclusion probability. Otherwise, specific versions of the usual statistics methods should be used. It is always risky to communicate data that one cannot process correctly using classical statistics methods. Hence, for avoiding this risk, it is advisable that the multistage sampling design be a self-weighting one. It is worth noting that self-weighted multistage sampling also allows balancing workload among the high-level units, which, again, may be interesting from a logistical point of view.

## Declaration of Competing Interest

The author declare that he has no known competing for financial interests or personal relationships that could have appeared to influence the work reported in this paper.
